# Control of a COVID-19 outbreak in a nursing home by general screening and cohort isolation in Germany, March to May 2020

**DOI:** 10.2807/1560-7917.ES.2021.26.1.2001365

**Published:** 2021-01-07

**Authors:** Manuel Krone, Annette Noffz, Elisabeth Richter, Ulrich Vogel, Michael Schwab

**Affiliations:** 1 Institute for Hygiene and Microbiology, University of Würzburg, Würzburg, Germany; 2 Buergerspital Foundation, Würzburg, Germany

**Keywords:** COVID-19, SARS-CoV-2, nursing, infection control

## Abstract

Elderly care facilities have become a major focus of coronavirus disease (COVID-19) control. Here, we describe an outbreak of COVID-19 in a nursing home in Germany from 8 March to 4 May 2020 (58 days), and the effect of an intervention of general screening and cohort isolation. COVID-19 cases among residents and staff were recorded on a daily basis from the first positive SARS-CoV-2 test from a resident on 8 March 2020, until 4 May 2020 when the last staff member was classified COVID-19 negative. Eighty of 160 residents (50%) and 37 of 135 staff members (27%) tested positive for SARS-CoV-2. Twenty-seven of the 80 residents were asymptomatic but tested positive during the first general screening. Cohort isolation of SARS-CoV-2 positive residents by reorganising the facility proved to be a major effort. After the intervention, four further asymptomatic residents tested positive in follow-up screenings within a period of 6 days, and were possibly infected prior to the intervention. Thereafter, no further infections were recorded among residents. The described outbreak was controlled by implementing general screening and rigorous cohort isolation, providing a blueprint for similar facilities.

## Introduction

Coronavirus disease (COVID-19) is a disease caused by the severe acute respiratory syndrome coronavirus 2 (SARS-CoV-2). It was first described in December 2019 in Wuhan, China, and has since spread around the world. By 27 October 2020, the number of worldwide cases had reached 43.5 million, with 1.2 million deaths, resulting in an average case fatality rate (CFR) of 2.7% [1]. Due to under-reporting of asymptomatic and oligosymptomatic cases, and symptomatic cases that have not undergone PCR testing, the number of cases is underestimated and the CFR may be overestimated. The degree of underestimation varies due to different case definitions and testing strategies used by different countries [2]. The median incubation time of COVID-19 is around 5 days but may range from 2–12 days after infection [3]. SARS-CoV-2 is transmitted by both respiratory droplets (> 5 µm) and contact with respiratory secretions. The contribution of aerosols (particles ≤ 5 µm which can be spread over larger distances) is not yet clear [4].

The first COVID-19 case in Germany was confirmed on 27 January 2020 [5]. As the epidemic progressed in Germany, the distribution of COVID-19 in different populations changed. There were only a few cases in the ≥ 80 years age group at the beginning of the epidemic [5], which changed during March and April. The ≥ 80 years age group, and to a greater extent the ≥ 90 years age group, have, as of May 2020, been overrepresented regarding both incidence and CFR [6]. The high attack rate in older adults in Germany, where 64% of COVID-19-related deaths occurred in the ≥ 80 years age group, is similar to other European countries (65% in the United Kingdom (UK) [7] and 57% in Italy [8] for the ≥ 80 years age group, and 71% in France for the ≥ 75 years age group) [9], but more pronounced compared with the United States (US) (58% of COVID-19-related deaths in the ≥ 75 years age group) [10] and China (20% of COVID-19-related deaths in the ≥ 80 years age group) [11]. The median age of COVID-19-related deaths in Germany up until May 2020 was 82 years [6], and 26% of COVID-19 cases in the ≥ 80 years age group died [12]. In the ≥ 80 years age group, relevant comorbidities were reported in 80% of deaths.

Alarming COVID-19 outbreaks in nursing homes have been reported from all around the world, where up to 85% of residents have been infected and up to 30% have died [13-17]. In a large facility in New York, 98 deaths linked to COVID-19 have been reported [14]. Until 25 May 2020, 45% of all German COVID-19-related deaths occurred in elderly people living in nursing homes or similar settings [6].

Important factors promoting the spread of the virus in facilities are the lack of personal protective equipment (PPE), lack of specially trained staff, difficulties with SARS-CoV-2 testing of staff and residents and residents often being unable to comply with instructions such as wearing masks or keeping distance [13,18,19]. Asymptomatic SARS-CoV-2 carriage was underestimated at the beginning of the epidemic, and was described in up to 57% of elderly persons who tested positive, which is likely to promote spread of the virus [16,20].

## Outbreak detection

On 8 March 2020, the first positive SARS-CoV-2 PCR test, performed on a resident who had been symptomatic since 4 days, was reported by the laboratory to a nursing home in Bavaria as well as to the local health authorities. SARS-CoV-2 testing in nursing homes was performed by a mobile testing ordering service of the Bavarian Association of General Practitioners and could only be ordered for symptomatic residents and staff until 23 March.

In this study, we describe a large outbreak of COVID-19 in a nursing home in Germany and elucidate how it was effectively controlled by general screening and rigorous set-up of a cohort isolation area.

## Methods

### Study design and data collection

In this observational study, we analysed the effect of general screening and structural intervention on the development of new COVID-19 cases in a nursing home. COVID-19 cases among residents and staff were recorded on a daily basis from the first positive SARS-CoV-2 test from a resident on 8 March 2020, until 4 May 2020 when the last staff member was classified COVID-19 negative. COVID-19 infections before and after the intervention (25–28 March 2020) were compared descriptively.

### Study setting and population

**Figure 1:**
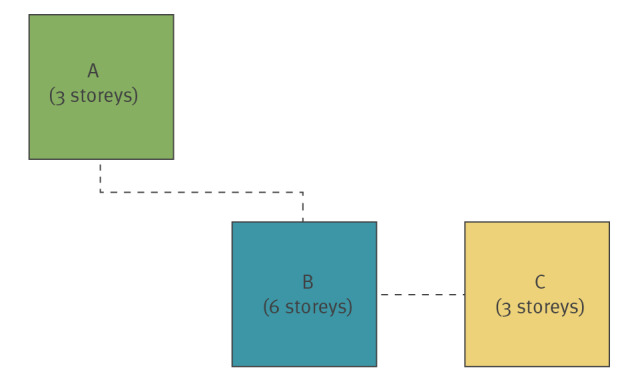
Overview of the nursing home’s three buildings A, B and C after separation into COVID-19 negative and COVID-19 positive isolation areas

In this non-controlled, retrospective observational study, we analysed the effect of a coordinated intervention on the development of a COVID-19 outbreak in a nursing home. The nursing home consists of three buildings - building A (three-storeys containing 45 beds), building B (six storeys containing 105 beds) and building C (three storeys containing 24 beds) (see Figure 1 for an overview of the location and buildings). In total, there are 174 available beds in 40 two-bed and 94 single-bed rooms. At the beginning of the study, 160 residents between their early sixties and aged up to over 100 years old (average age 86 years) were living at the home.

COVID-19: coronavirus disease.

COVID-19 negative areas were declared in buildings A and C, and on floors 1 and 3 of building B.

COVID-19 positive areas were established in building B on floors 0, 2, 4, and 5.

Building A was the least affected area with only one resident testing positive during the course of the outbreak.

The dotted lines represent the connections between buildings.

### SARS-CoV-2 testing and discharge of SARS-CoV-2-positive residents from quarantine

SARS-CoV-2 testing was performed on oropharyngeal swabs taken by healthcare workers from a mobile testing ordering service until 23 March, and from 24 March by the consultant of the geriatric rehabilitation unit. The swabs were analysed by different laboratories serving the facility and the public health office using reverse transcription PCR (RT-PCR). COVID-19 cases were defined by a positive SARS-CoV-2 test. Cases were counted on the day of their positive SARS-CoV-2 result as sampling dates were not reliably available during the first phase of the outbreak. Residents and staff were classified recovered after a minimum of 14 days after symptom onset after they were asymptomatic for at least 2 days, and after two consecutive tests for SARS-CoV-2 taken at least 24 hours apart proved to be negative.

### Outbreak control

Residents with respiratory symptoms, fever or a positive SARS-CoV-2 test were separated from other residents into single rooms and hygiene precautions were intensified: residents were not allowed to leave the room, and staff caring for residents had to wear PPE consisting of a respirator mask, safety goggles, protective gowns, and gloves. According to the recommendations of the Robert Koch Institute (German national public health institute) [21], staff who tested positive and their close contacts received a self-isolation order by the local public health office, which conducted contact tracing. A visitor ban was announced in the facility on 9 March, 12 days before a general visitor ban was declared in all nursing homes in the state of the Bavaria [22]. External healthcare workers such as physiotherapists were banned from the facility.

After the measures had failed to control the outbreak despite sufficient availability of PPE and staff regularly trained in PPE usage, the mayor declared the home a state of emergency on 16 March. The consultant of the geriatric rehabilitation unit was made solely responsible for primary medical care and SARS-CoV-2 testing of the residents. As testing resources, which had previously not been available in sufficient amounts, became available, an intervention took place consisting of a general SARS-CoV-2 screening of all residents in the evening of 24 March and all staff members on 25 March. The first results of the general screening were available on 26 March, while the latest reports arrived on 28 March. On 28 March, the cohort of residents who tested positive were isolated. From 24 March, the consultant took all swabs, instead of the mobile testing ordering service.

On 28 March, building B (Figure 1) was divided into a COVID-19 positive area located on floors 0, 2, 4, and 5, and a COVID-19 negative area on floors 1 and 3. Buildings A and C were made a COVID-19 negative area (Figure 1). Staff were divided into permanent and physically separate teams for each area. Access to the two areas, supply and disposal was ensured by separate staircases and lifts. Relocation of the residents and their belongings, and cleaning, disinfecting and preparing the rooms was carried out by a joint effort of 145 people and lasted from 06:00 until 01:30 the next day.

To prevent a resurgence of the outbreak in the COVID-19 negative area, SARS-CoV-2 testing was performed on all previously COVID-19 negative residents on 29 March, 3 April, 7 April and 14 April, with one median incubation period in between testing dates. Additionally, symptomatic residents were tested directly after symptom onset. As soon as any resident tested positive, they were moved to the COVID-19 positive area. Recovered COVID-19 positive residents stayed in the COVID-19 positive area despite limited evidence for immunity, as no human reinfection had yet been confirmed [23]. Droplets, direct contact and possibly aerosols were assumed to be the primary mode of transmission of SARS-CoV-2 [4]. As testing capacity was still limited, no environmental samples were tested for SARS-CoV-2 in the facility.

Low-threshold testing of symptomatic staff continued after the intervention. In addition, all asymptomatic staff members on duty were tested on 2 April, followed by a second test on 7-8 April and a third test on 14-18 April. Testing intervals for staff and residents were chosen with about one median incubation period between testing dates. Both permanently employed staff and temporary staff were included in the testing. Due to the rota system, testing had to be expanded over several days to include all staff members. During the period studied, approximately 1,100 SARS-CoV-2 tests were performed on residents and staff.

In total, 34 additional staff members from other facilities had supported the nursing home’s core staff since 15 March. Before the outbreak, residents were treated by their individual general practitioner (GP), and up to 30 different GPs were active in the home. From 2 April, three GPs were assigned to take care of all residents inside the home, 7 days a week by decree. In contrast to reports of PPE shortages worldwide [17-19], there was always enough PPE available to comply with hygiene precautions.

### Ethical statement

Ethical approval was not required for this study as no individual data were used in this study.

## Results

### Description of the outbreak

We describe an outbreak of COVID-19 in a nursing home affecting 80 of 160 residents (50%) and 37 of 135 staff members (27%). The case fatality ratio in residents was 31% (25/80), and none of the staff members died. The outbreak lasted 58 days, from 8 March to 4 May 2020.

**Figure 2:**
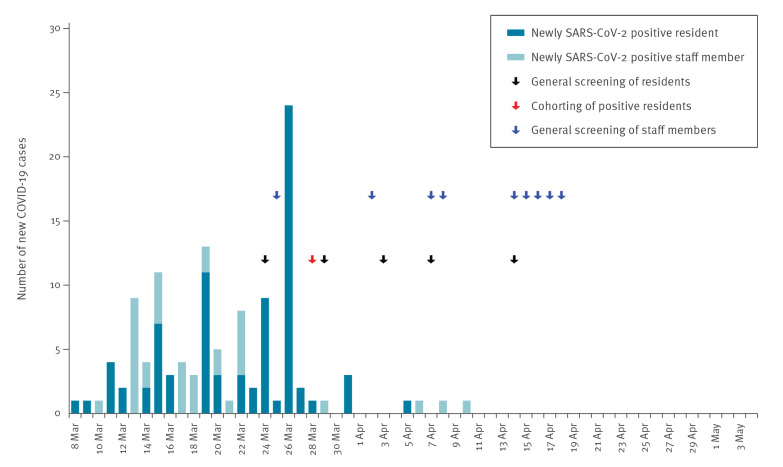
Number of newly diagnosed COVID-19 cases among nursing home residents and staff by test result date, Wuerzburg, Germany, 8 March–4 May 2020 (n = 160 residents, n = 135 staff)

COVID-19: coronavirus disease; SARS-CoV-2: severe acute respiratory syndrome coronavirus 2.

After the first positive SARS-CoV-2 test of a symptomatic resident on 8 March, an additional 48 symptomatic residents were diagnosed with COVID-19 in the following 17 days prior to the intervention. It was decided that complete SARS-CoV-2 testing of 111 asymptomatic residents would be performed, and this was conducted on 24 March when sufficient testing capacities became available. The testing revealed 27 asymptomatic SARS-CoV-2 positive residents (24% of all 111 asymptomatic residents). None of these residents became symptomatic. In the first follow-up screening on 29 March, three further asymptomatic COVID-19 cases were diagnosed. The last COVID-19 case reported in a resident was diagnosed during the second follow-up screening on 3 April, 6 days after the intervention (Figure 2).

**Figure 3:**
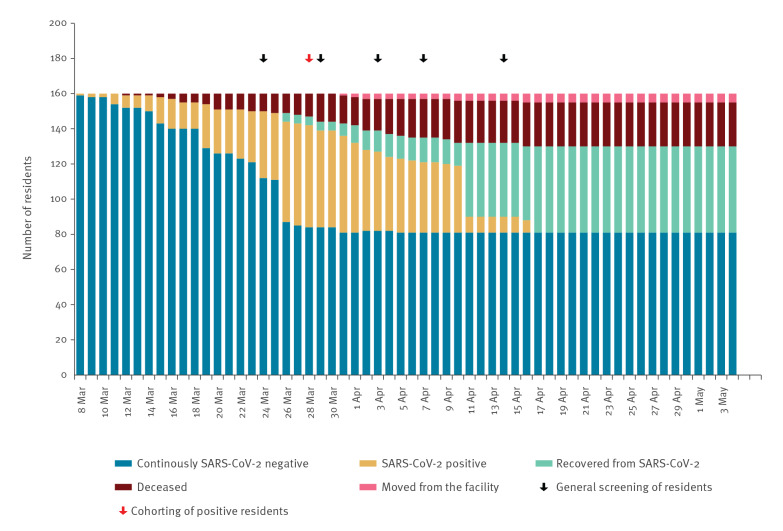
SARS-CoV-2 testing status and outcome for nursing home residents by date, Wuerzburg, Germany, 8 March–4 May 2020 (n = 160)

COVID-19: coronavirus disease; SARS-CoV-2: severe acute respiratory syndrome coronavirus 2.

The last SARS-CoV-2 positive resident was classified SARS-CoV-2 negative on 17 April (Figure 3).

Residents were classified positive on the day their first positive SARS-CoV-2 test was reported, and classified recovered after a minimum of 14 days after symptom onset after they were asymptomatic for at least 2 days, and after two consecutive tests for SARS-CoV-2 taken at least 24 hours apart proved to be negative.

Eighty residents remained SARS-CoV-2 negative. Eighty residents tested SARS-CoV-2 positive, 55 recovered and 25 died.

During the outbreak period, five residents moved home or to another facility.

While the majority of the COVID-19 cases occurred in residents of buildings B (66/98) and C (13/24), only 1 of 38 residents living in building A tested positive for SARS-CoV-2. Infected residents were identified on all floors of buildings B and C.

**Figure 4:**
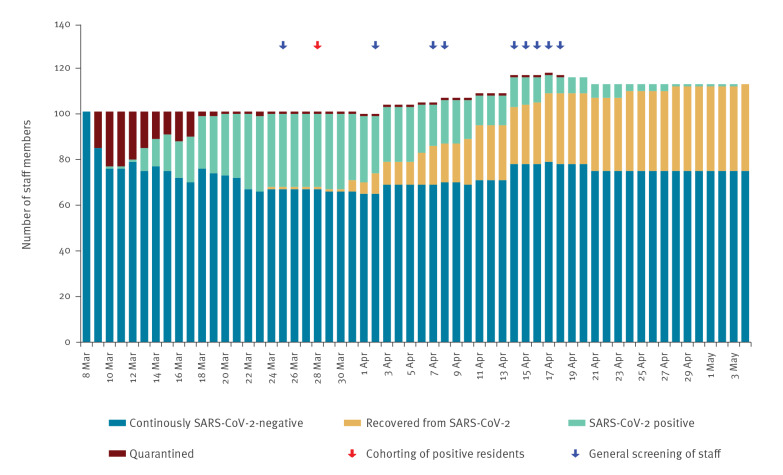
SARS-CoV-2 testing status and outcome for nursing home staff by date, Wuerzburg, Germany, 8 March–4 May 2020 (n = 135)

In total, 37 of 135 (27%) staff members working at the facility were infected with SARS-CoV-2. A total of 36 of 101 (36%) staff members working at the facility before the outbreak tested positive, whereas 1 of 34 (3%) temporary staff was infected. Temporary staff were needed to replace infected permanent staff and their close contacts among staff in quarantine. The first COVID-19 positive staff member was diagnosed on 10 March. Until 26 March, a total of 33 staff members were diagnosed with COVID-19. Four further staff members were diagnosed after the intervention, the last on 10 April. No member of staff died during the observation period. The last staff member was classified SARS-CoV-2 negative on 4 May while still hospitalised (Figure 4).

SARS-CoV-2: severe acute respiratory syndrome coronavirus 2.

Staff members were classified positive on the day their first positive SARS-CoV-2 test was reported, and classified recovered after a minimum of 14 days after symptom onset after they were asymptomatic for at least 2 days, and after two consecutive tests for SARS-CoV-2 taken at least 24 hours apart proved to be negative. Staff members with close contact to SARS-CoV-2 positive residents or staff members were quarantined if no adequate PPE was worn.

Ninety-eight staff members remained SARS-CoV-2 negative. Thirty-seven staff members tested SARS-CoV-2 positive, all recovered.

The implementation of separation and screening was associated with a considerable reduction in new infections, and finally the termination of the outbreak.

## Discussion

This report describes an outbreak in a nursing home with 49 SARS-CoV-2 positive residents and 33 SARS-CoV-2 positive staff within 18 days. This rapid course with a high attack rate among residents and staff resembles previously described outbreaks in nursing homes and underlines the perils for these facilities associated with COVID-19 [14-16]. We further describe an effective control strategy for a COVID-19 outbreak in a nursing home.

General screening revealed that 24% of the asymptomatic residents were infected by the virus. Data from nursing, hospital and community clusters suggest that asymptomatic or pre-symptomatic persons play an important role in SARS-CoV-2 transmission [20,24,25]. It is likely that control of the outbreak, where all conventional measures such as symptom-based testing, visitor ban, intensified hygiene measures and skilled usage of sufficient PPE had been exhausted, was only possible by detecting those asymptomatic individuals through rigorous screening, and followed by isolation. General screening has been proposed as an important tool to control those asymptomatically infected with SARS-CoV-2 before the onset of disease [16,20] as virus concentrations in upper respiratory secretions are high 2 days before symptom onset [24]. In contrast to another study [16], none of the asymptomatic SARS-CoV-2 positive residents in this report went on to develop symptoms, which was a surprising finding given the age and co-morbidities of the residents.

In addition to general screening and isolating residents who tested positive, as described in earlier literature [15,16], rigorous cohort isolation was associated with the termination of the described outbreak. Cohorting in nursing homes has been reported as a control strategy in influenza outbreaks [26]. The last four SARS-CoV-2 positive residents identified in the COVID-19 negative area after the intervention were most likely already infected at the time of the intervention given the typical long incubation of this disease [3]. No further cases were detected in residents afterwards, suggesting the intervention was successful. Furthermore, the authors recommend that every nursing home should have a pandemic preparedness plan.

The cost of the intervention was notable, as more than 140 staff worked over 18 hours to execute the division of the sectors. In addition, testing costs of more than EUR 50,000 were incurred. This needs to be taken into account in comparable situations. However, the intervention as a whole is suggested to be cost-effective when weighed against the devastating impact of the outbreak on resident and staff health, the huge cost of temporary staff and the loss of reputation of the nursing home, which would have been aggravated had the outbreak not been stopped.

There were problems in managing the outbreak that may have also been experienced by other facilities during the first wave of the COVID-19 pandemic. As seen in many countries [17,18], initially, there was a major lack of testing capacity restricting tests to only symptomatic residents and staff. Given the high proportion of asymptomatic residents with SARS-CoV-2 positive test results, this likely supported the spread of the disease. The absence of infected and quarantined staff lead to understaffing, which has also been reported elsewhere [14,17,18]. Medical care of nursing home residents in Germany is the responsibility of the GP selected by the residents or their family. Thus, up to 30 different physicians were responsible for the residents. This impeded a coordinated outbreak response, which was corrected by the local authorities once the state of emergency legislation was passed by having only three GPs taking care of all residents’ medical issues. Delegating the testing to one person, the consultant of the geriatric rehabilitation unit, also facilitated the process considerably compared with the distributed responsibilities at the beginning of the outbreak. While PPE was always sufficiently available in this nursing home, the unavailability of PPE, which was a worldwide constraint [17-19], may additionally promote staff contamination and infection. Future pandemic preparation should include sufficient stockpiling of PPE, a simple measure of protection [15,18].

It is striking that COVID-19 hit this nursing home so dramatically even though it employs a large proportion of skilled, permanently employed staff participating in annual infection control training. In contrast to the COVID-19 outbreak in a Quebec nursing home where many staff had left work [27], staff at this institution were highly motivated to contribute to controlling the outbreak. Next to the sufficient availability of SARS-CoV-2 tests and massive support by local public institutions to implement the cohorting, workforce stability and loyalty appear to have been the key to outbreak control.

Universal SARS-CoV-2 testing on all nursing home residents and staff after the detection of one COVID-19 case in a facility has meanwhile become part of the European Centre for Disease Prevention and Control (ECDC) and the Centers for Disease Control and Prevention (CDC) recommendations. Additional recommendations are dedicated areas to cohort SARS-CoV-2 positive residents, and sufficient PPE stockpiling [28-30]. Data from the described outbreak show the importance of these combined measures in quickly and efficiently terminating a COVID-19 outbreak in a nursing home. COVID-19 outbreaks are to be expected in similar facilities worldwide, and preparing for these outbreaks is vital. A relevant proportion of COVID-19 deaths occur in nursing homes [6]. Thus, quickly terminating SARS-CoV-2 outbreaks in such facilities contributes considerably to reducing COVID-19 mortality in the general population in Europe and the Americas. Despite the effective intervention for terminating an outbreak in nursing homes presented here, appropriate infection prevention and control measures to prevent the entrance of SARS-CoV-2 into nursing homes are of extreme importance. Additionally, watching for typical and atypical symptoms of COVID-19 in residents and staff are vital in detecting and controlling virus spread as soon as possible.

Isolation in nursing homes has a negative impact on both residents’ mental health [31] and general healthcare, as observed in this facility. While it is not possible to completely avoid the negative impact in the situation of a COVID-19 outbreak, a strategy outlining the quick termination of a COVID-19 outbreak may enable nursing homes to more quickly allow visitors again and prevent the negative health impact of loneliness.

The outbreak description has several limitations. Though the outbreak stopped after the intervention, causality cannot be proven as a bundle of interventions were employed. The observation was retrospective and lacked a control group. The source case of the COVID-19 outbreak remains unclear but it may, according to the timing, have been an unknown traveller, either staff or visitor, returning from an affected area with virus circulation at that time such as Italy who introduced the virus into the facility. The majority of the COVID-19 infections in Bavaria at the start of the pandemic wave had this source of infection [32].

## Conclusion

This description of a successful control of a COVID-19 outbreak in a nursing home may support others in similar efforts. The combination of general SARS-CoV-2 screening and consistent cohorting of residents who tested positive or negative proved to be a laborious but powerful approach to outbreak control. Skilled and motivated staff, focused medical responsibilities, vigorous support by the community in the frame of emergency state legislation and structures, and sufficient PPE and testing capacities are crucial for controlling an outbreak in this vulnerable setting.
